# Methanol steam reforming performance and catalytic mechanism of bimetal-loaded cerium oxide-based thermal catalysts

**DOI:** 10.1039/d6ra02054b

**Published:** 2026-04-22

**Authors:** Chi Zhang, Yongning Ma, Mingyuan Guo, Yuhao Yang, Xiaolong Li, Enzhou Liu

**Affiliations:** a College of Chemistry and Chemical Engineering, Shaanxi University of Science & Technology Xi'an 710072 China ynma@sust.edu.cn; b College of Chemistry and Materials Science, Weinan Normal University 714099 China; c School of Chemical Engineering, Northwest University Xi'an 710069 PR China liuenzhou@nwu.edu.cn

## Abstract

An activity evaluation was performed for the replacement of the noble metal Pt with non-precious metal active species while maintaining catalytic performance in methanol steam reforming. In this study, bimetallic cerium oxide-supported catalysts (Pt_*x*_M_10−*x*_/N-CeO_2_) were prepared using Pt and non-precious metals. Among these catalysts, the Pt–Fe system exhibited the highest reforming performance. At 180 °C, the H_2_ production rate reached 17.29 mmol *g*_cat_^−1^·h^−1^, with a CO selectivity of 15.58% and methanol conversion of 1.07%. Results indicated that the introduction of Fe effectively increased the specific surface area and the amount of lattice oxygen on the catalyst surface while promoting the formation of abundant oxygen vacancies. Under optimal reforming conditions, Pt_5_Fe_5_/N-CeO_2_ showed the lowest energy consumption and the best reforming performance. Furthermore, based on the thermodynamic analysis of the methanol steam reforming reaction, the corresponding reaction pathways and catalytic mechanisms were proposed.

## Abstract 2

Bimetallic CeO_2_-supported catalysts were successfully prepared by partially replacing Pt with non-precious metals. At 180 °C, they exhibited excellent performance in methanol steam reforming for hydrogen production.

## Graphical abstract

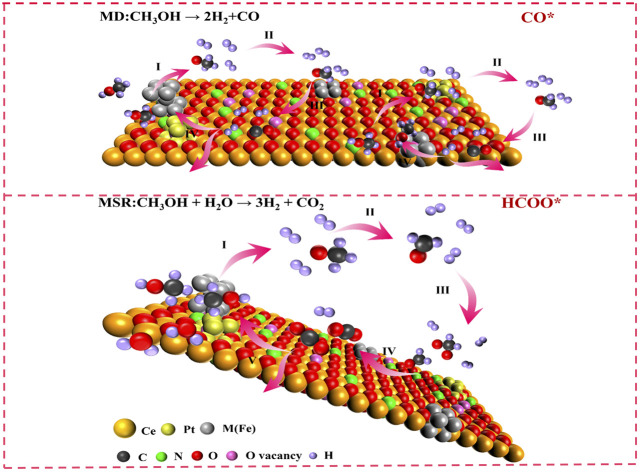

## 1 Introduction

Transition metal oxides (TMOs) have been widely studied for their application as catalysts in low-temperature methanol steam reforming (LT-MSR) reaction due to their low cost and considerable catalytic activity.^1–3^ They have the advantages of variable valence states and tunable orbital occupancy in redox reactions, making them the most promising candidates to replace precious metals.^4–6^ The reported catalytic systems mainly include Ce-, Mo-, Cu- and Ti-based catalysts and multicomponent composite systems.^7,8^ Among these, the ceria-based catalysts exhibit a reversible Ce^3+^/Ce^4+^ redox cycle, abundant oxygen vacancies within the structure, and strong metal–support interactions, which synergistically regulate the surface electronic structure. In addition, the exposure of different crystal facets further enhances surface reactivity. These combined effects enable ceria-based catalysts to display excellent redox properties and generate a large amount of highly active surface oxygen species.^9–11^ Thus, these catalysts show excellent redox activity, strong thermal stability, high surface reactive oxygen concentration and structural stability.^12–14^

Studies show that transition metal oxide-loaded noble metal catalysts show great potential in methanol steam reforming (MSR) reactions, and their catalytic properties are sensitive to the structural features of the transition metal oxide interface, which are generally determined by the properties of the support and loaded metal.^15–17^ Compared with other metal (Au, Ag, Pd, Pt, Ru, Rh, and Ir)-loaded catalysts, cerium oxide-loaded Pt atomic catalysts exhibit significant advantages due to their high catalytic properties, abundance, and easy and affordable preparation process.^18–20^ Among transition metals, Fe, Ni, and Zn exhibit considerable catalytic activity and are much cheaper than Pt. Therefore, identifying alternative metals to replace noble metal loading without compromising catalytic performance is the focus of this study.

In this study, N-doped CeO_2_ nanorods were prepared by thermal condensation. Under the condition that the Pt loading was set to 0.5 wt% and the Pt : M molar ratio was fixed at 1 : 1, a series of bimetallic Pt–M (M = Fe, Ni, Zn, Mg, Al, and Cu) catalysts supported on N-CeO_2_ were prepared *via* an impregnation method, followed by drying and thermal treatment. Then, by adjusting the atomic ratio of Pt to non-precious metals (by adjusting the molar ratio), thermal catalysts with different atomic ratios (Pt_*x*_M_10−*x*_/N-CeO_2_) were prepared by calcining the mixture at high temperatures. The crystal structure, microstructure, specific surface area, oxygen vacancy concentration, surface chemical composition, elemental state and chemical bond properties of the prepared thermal catalysts were investigated. In addition, their catalytic performance in methanol steam reforming was evaluated, and the possible reaction pathways and mechanisms of the Pt_*x*_M_10−*x*_/N-CeO_2_ catalysts were discussed.

## 2 Experiments

### 2.1 Materials

Cerium nitrate (Ce(NO_3_)_3_, AR) was purchased from Tianjin Damao Chemical Reagent Co., Ltd Sodium hydroxide (NaOH, AR) was obtained from Shanghai Macklin Biochemical Technology Co., Ltd Ethylenediamine (C_2_H_8_N_2_, AR) was supplied by Xilong Scientific Co., Ltd Chloroplatinic acid (H_2_PtCl_6_·6H_2_O, AR) was purchased from Shanghai Aladdin Biochemical Technology Co., Ltd Copper sulfate (CuSO_4_, AR), aluminum nitrate (Al(NO_3_)_3_, AR), nickel nitrate (Ni(NO_3_)_2_, AR), iron nitrate (Fe(NO_3_)_3_, AR), zinc nitrate (Zn(NO_3_)_2_, AR), magnesium nitrate (Mg(NO_3_)_2_, AR), anhydrous ethanol (AR), anhydrous methanol (AR), and sodium d-isoascorbate (C_6_H_7_NaO_6_, AR) were all obtained from Aladdin Reagent Co., Ltd Deionized water was prepared in the laboratory.

### 2.2 Characterization

X-ray photoelectron spectroscopy (XPS, Kratos AXIS Nova) was employed to analyze the surface elemental composition and chemical states of the samples. The N_2_ adsorption–desorption isotherms of the catalysts were measured using a surface area and pore structure analyzer (Beckman LS-230). The crystal structures of the samples were characterized by X-ray diffraction (XRD, D8 Advance, Bruker, Germany). Fourier transform infrared spectroscopy (FT-IR, VECTOR2, Bruker, Germany) was performed to identify the surface functional groups. The morphology and microstructure were examined using a field-emission scanning electron microscope (SEM, SU8100, Hitachi, Japan). Raman spectra were recorded on a Raman spectrometer (LabRAM Aramis, HORIBA, France). The specific surface area was calculated by the Brunauer–Emmett–Teller (BET) method. Cyclic voltammetry (CV) curves were obtained using an electrochemical workstation (CHI660D, Chenhua Instruments, Shanghai, China).

### 2.3 Evaluation of catalytic performance

Catalytic performance in methanol steam reforming (MSR) for hydrogen production was evaluated in a fixed-bed quartz tubular reactor. A total of 200 mg of the catalyst (180–200 mesh) was loaded into the reactor. The catalyst was first reduced in a H_2_/Ar mixed gas at a flow rate of 35 mL min^−1^ for 1 h under specified conditions. After reduction, the reactor was cooled to the desired reaction temperature under a 35 mL min^−1^ Ar atmosphere, and the fixed-bed reaction program was initiated. A CH_3_OH/H_2_O mixture with a molar ratio of 1 : 1.2 was fed into a vaporization chamber using a micro-pump at a flow rate of 0.1 mL min^−1^. The vaporizer temperature was maintained at 200 °C to ensure complete evaporation of the liquid mixture into the gas phase. The corresponding weight hourly space velocity (WHSV) was 26 h^−1^. The vapor was then carried into the reactor by Ar at a flow rate of 35 mL min^−1^. The outlet gas mixture was first passed through a cold trap to condense the unreacted components, and the remaining gas was subsequently analyzed online using a GC9790II gas chromatograph equipped with a chromatographic column (approximately 100 °C) and a thermal conductivity detector (TCD, approximately 130 °C), with 99.99% Ar as the carrier gas. The concentrations of H_2_, CO_2_, CH_4_ and CO were monitored. Methanol conversion was calculated based on the carbon balance. For catalytic activity evaluation, measurements at each temperature were repeated three times, and the average values were reported.1
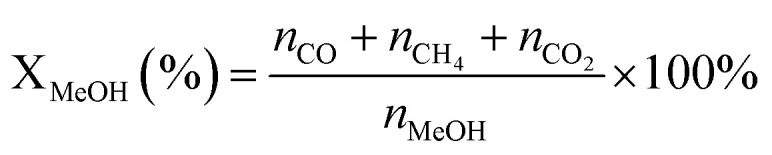
2
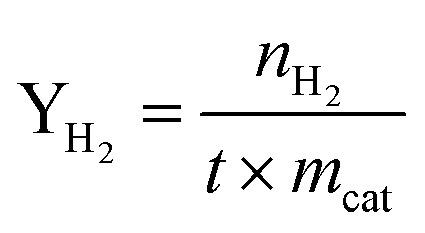
3
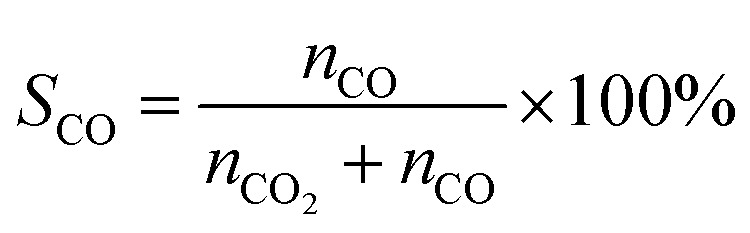
X(MeOH): methanol conversion (%). Y(H_2_): hydrogen production rate (mmol·*g*_cat_^−1^·h^−1^); *n*: molar amount of gas (mmol); *m*_cat_: mass of the catalyst (*g*); *t*: reaction duration (h); *S*: selectivity towards by-products CO (%).

### 2.4 Preparation of the N-CeO_2_ and Pt_*x*_M_10−*x*_/N-CeO_2_ catalysts

N-CeO_2_ nanorods were prepared as follows: first, 2.605 g of cerium nitrate and 5 g of sodium hydroxide were dissolved in 80 mL of deionized water and stirred at 80 °C for 1 h. During the stirring process, 720 µL of ethylenediamine aqueous solution (75 vol%) was added dropwise. The resulting mixture was then transferred to a Teflon-lined autoclave and maintained at 120 °C for 24 h. After the reaction, the product was collected, ultrasonically washed, centrifuged, and freeze-dried. The obtained solid was then calcined in a muffle furnace at 450 °C for 5 h to yield yellow CeO_2_ nanorods. Subsequently, 0.5 g of CeO_2_ nanorods and 0.198 g of sodium d-isoascorbate were dispersed in 200 mL of deionized water and stirred at room temperature for 5 h. After ultrasonic washing, the N-doped CeO_2_ nanorods were obtained.

Pt_5_M_5_/N-CeO_2_ nanorods were prepared as follows: The Pt_5_M_5_/N-CeO_2_ catalyst was prepared by the impregnation method. The specific procedure is as follows: firstly, 0.35 g of N-CeO_2_ nanorods, 0.00226 g of CuSO_4_·5H_2_O (0.00263 g of Ni(NO_3_)_2_·6H_2_O, 0.00269 g of Zn(NO_3_)_2_·6H_2_O, 0.00232 g of Mg(NO_3_)_2_·6H_2_O, 0.00339 g of Al(NO_3_)_3_·9H_2_O, 0.00366 g of Fe(NO_3_)_3_·9H_2_O), and 938 µL of chloroplatinic acid solution (5 g L^−1^) were dispersed in 60 mL of deionized water under continuous stirring. The mixture was stirred at room temperature for 5 h to ensure sufficient contact. Subsequently, the product was ultrasonically washed, centrifuged, and freeze-dried to obtain a gray flocculent solid. Finally, the obtained product was transferred to a crucible and calcined in a muffle furnace at 300 °C for 1 h. The resulting solid powders were denoted as Pt_5_Cu_5_/N-CeO_2_, Pt_5_Al_5_/N-CeO_2_, Pt_5_Ni_5_/N-CeO_2_, Pt_5_Fe_5_/N-CeO_2_, Pt_5_Zn_5_/N-CeO_2_, and Pt_5_Mg_5_/N-CeO_2_ nanorods (Pt loading was 0.5 wt% and the molar ratio of Pt atoms to other metal atoms was 1 : 1).

**Fig. 1:**
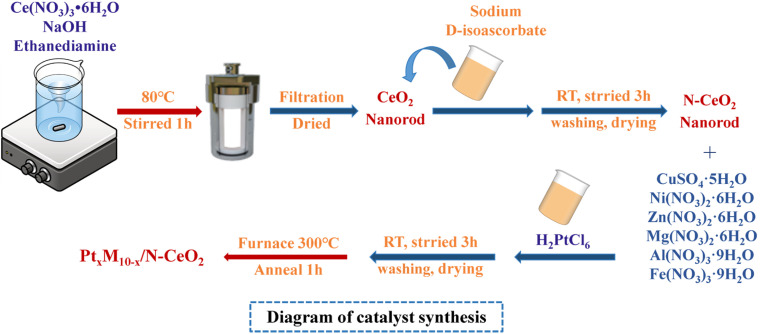
Preparation process of Pt_*x*_M_10−*x*_/N-CeO_2_.

Pt_*x*_Fe_10−*x*_/N-CeO_2_ nanorods were prepared as follows: The Pt_*x*_Fe_10−*x*_/N-CeO_2_ catalyst was also prepared by the impregnation method. The specific procedure is as follows: firstly, 0.35 g of N-CeO_2_ nanorods, 0.00658 g (0.00071 g, 0.00512 g, 0.00219 g, and 0.00366 g) of Fe(NO_3_)_3_·9H_2_O, and 188 µL (1690 µL, 563 µL, 1314 µL, and 938 µL) of chloroplatinic acid solution (5 g L^−1^) were dispersed in 60 mL of deionized water under continuous stirring. The mixture was stirred at room temperature for 5 h. After the reaction, the products were collected by filtration, washed with ethanol and deionized water, and then freeze-dried to obtain a gray flocculent solid. The dried samples were transferred to a crucible and calcined in a muffle furnace (heating rate of 5 °C min^−1^) at 300 °C for 1 h. The resulting solid powders were denoted as Pt_1_Fe_9_/N-CeO_2_, Pt_9_Fe_1_/N-CeO_2_, Pt_3_Fe_7_/N-CeO_2_, Pt_7_Fe_3_/N-CeO_2_, and Pt_5_Fe_5_/N-CeO_2_ nanorods (where *x*:10 − *x* is the molar ratio of Pt to Fe). The preparation process is illustrated in Fig. 1.

## 3. Results and discussion

### 3.1 Morphology and structure analysis of the Pt_*x*_M_10−*x*_/N-CeO_2_ catalysts

**Fig. 2:**
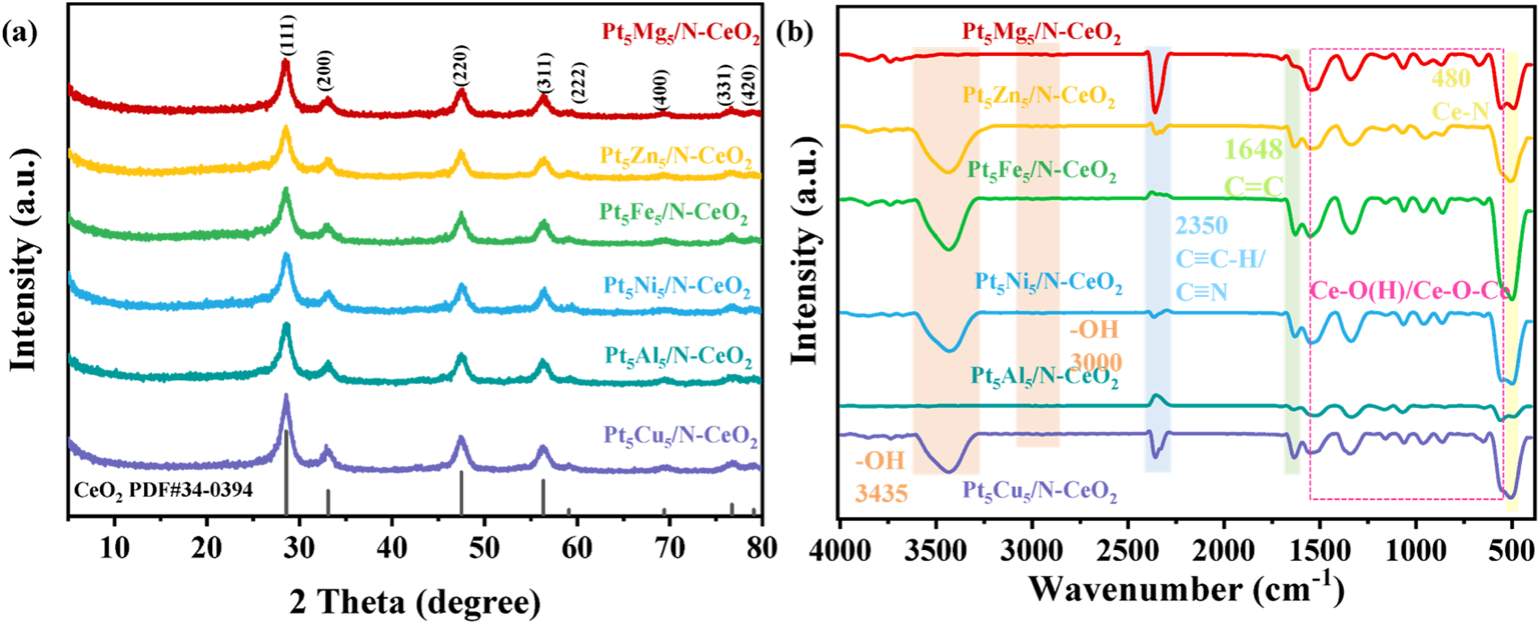
(a) XRD patterns and (b) FT-IR spectra of the Pt_5_M_5_/N-CeO_2_ catalysts.

Fig. 2a shows the XRD patterns of catalysts (Pt_5_Cu_5_/N-CeO_2_, Pt_5_Al_5_/N-CeO_2_, Pt_5_Ni_5_/N-CeO_2_, Pt_5_Zn_5_/N-CeO_2_, Pt_5_Fe_5_/N-CeO_2_ and Pt_5_Mg_5_/N-CeO_2_) prepared with 0.5 wt% Pt loading while keeping the molar ratio of Pt atoms to other metal atoms 1 : 1. Fig. 2a shows the XRD patterns of Pt_*x*_M_10−*x*_/N-CeO_2_ samples. All the samples exhibit diffraction peaks at 2*θ* = 28.5°, 33.1°, 47.4°, 56.3°, 59.0°, 69.4°, 76.6°, and 79.1°,^21,22^ which are attributed to the (111), (200), (220), (311), (222), (400), (331), and (420) crystal planes (PDF#34-0394), respectively. No characteristic peaks corresponding to the loaded metals were observed in the XRD patterns, which may be attributed to the low Pt loading (0.5 wt%) and the relatively low content of other metals, as well as their highly dispersed distribution within CeO_2_. Notably, significant differences in the (111) diffraction peaks are observed among the samples, suggesting that metal loading affects the crystal structure of CeO_2_ and may induce lattice distortion or crystallinity variations, resulting in different degrees of changes in the (111) plane.

The FTIR spectra of Pt_*x*_M_10−*x*_/N-CeO_2_ (M: Cu, Al, Ni, Zn, Fe, and Mg) samples are shown in Fig. 2b. The absorption peak at 3435 cm^−1^ corresponds to the stretching vibration of adsorbed water molecules on the sample surface. The peak observed at 2350 cm^−1^ can be attributed to the stretching vibration of C≡C–H or nitrile groups (C≡N). The absorption peak at 480 cm^−1^ is assigned to the Ce–N stretching vibration, indicating that N atoms have been successfully introduced into the sample and that new chemical bonds have been formed. The absorption peaks in the range of 510–1553 cm^−1^ correspond to the Ce–O stretching vibrations.^21,23^ It can be seen that the loaded metal elements have a negligible influence on the characteristic peaks of CeO_2_, and the product essentially maintains its original structure.

**Fig. 3:**
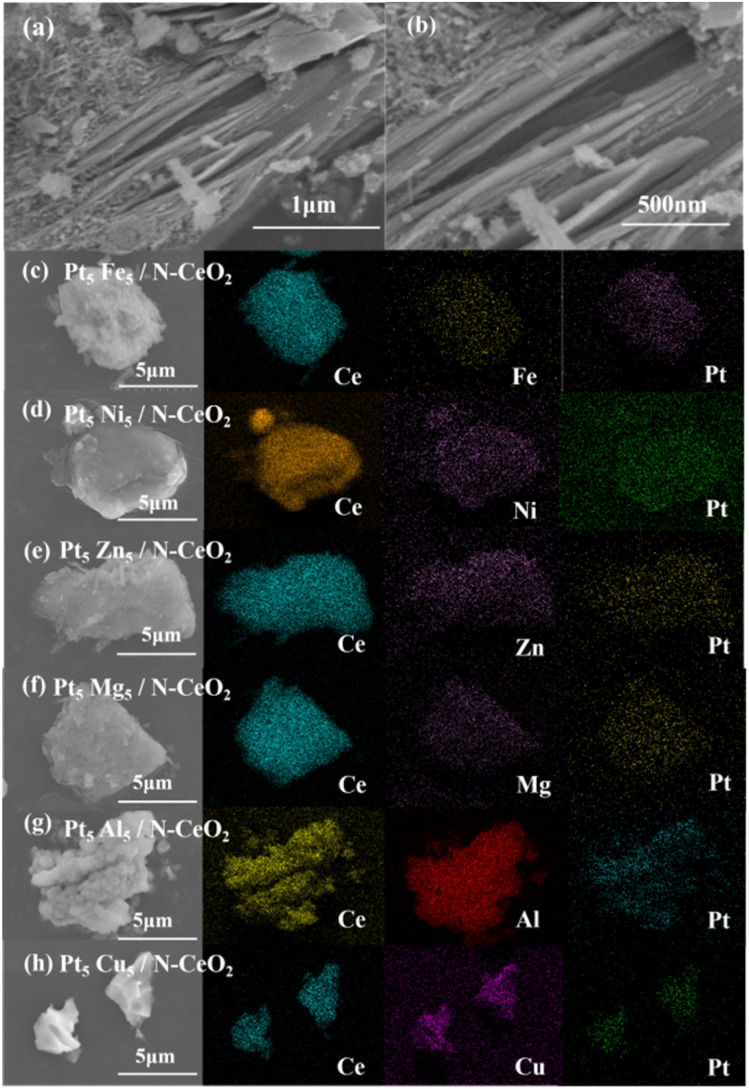
(a and b) SEM image of Pt_5_Fe_5_/N-CeO_2_. (c–h) SEM-EDS elemental mapping of Pt_5_M_5_/N-CeO_2_.

Fig. 3 shows the microscopic morphology and elemental distribution of the samples. Fig. 3a and b present the scanning electron microscopy (SEM) images of Pt_5_Fe_5_/N-CeO_2_. It can be observed that Pt_5_Fe_5_/N-CeO_2_ exhibits a block-like structure assembled from nanorods, with an overall size of approximately 6 µm, while the nanorods have an average diameter of about 10 nm. Some partially damaged regions can be observed on the surface of Pt_5_Fe_5_/N-CeO_2_, indicating a relatively rough surface. Such a rough surface is beneficial for increasing the specific surface area of the catalyst and promoting the dispersion of active components. Fig. 3c–h show the SEM-EDS mapping images of Pt_5_Fe_5_/N-CeO_2_, Pt_5_Ni_5_/N-CeO_2_, Pt_5_Zn_5_/N-CeO_2_, Pt_5_Mg_5_/N-CeO_2_, Mg_5_Al_5_/N-CeO_2_, and Pt_5_Cu_5_/N-CeO_2_, respectively. It can be observed that Fe, Ni, Zn, Mg, Al, and Cu are uniformly distributed on the surface of CeO_2_.

**Fig. 4:**
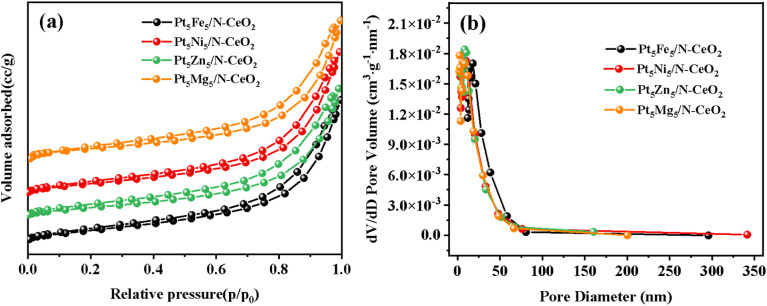
(a) N_2_ adsorption–desorption equilibrium curves of the Pt_5_M_5_/N-CeO_2_ thermal catalysts and their (b) pore size distribution curves.

Fig. 4 shows the N_2_ adsorption–desorption equilibrium curves and pore size distribution curves of Pt_5_Fe_5_/N-CeO_2_, Pt_5_Ni_5_/N-CeO_2_, Pt_5_Zn_5_/N-CeO_2_ and Pt_5_Mg_5_/N-CeO_2_. As shown in Fig. 4a, all the samples exhibit a type IV isotherm with H_3_ lag loop and rapid absorption under a low relative pressure. These results showed that the samples are characterized by the co-existence of mesopores and micropores. As shown in Table 1, the specific surface areas of each catalyst were calculated to be 142.827 m^2^ g^−1^, 133.858 m^2^ g^−1^, 129.307 m^2^ g^−1^ and 126.315 m^2^ g^−1^, respectively. The specific surface area of the samples varied from 126.315 to 142.8 m^2^ g^−1^, indicating that the type and shape of the loaded metal had an effect on surface area. Fig. 4b shows the pore size distribution curves of the samples, indicating that all the samples are mesoporous materials. The cumulative pore size distribution of the Pt_5_Fe_5_/N-CeO_2_ catalyst was calculated by the BJH fitting method. The pore size of the Pt_5_Fe_5_/N-CeO_2_ catalyst is approximately 8 nm, while the pore size of the other samples ranges from 6 to 8 nm. The experimental results demonstrate that large surface area and a high density of surface defects not only improve the dispersion of catalytically active species but also anchor Pt species, inhibiting their aggregation. In addition, the larger pore volume is conducive to facilitating the mass transfer of methanol and water molecules in the thermal catalytic reaction process to achieve better thermal catalytic activity.

**Table 1:** Specific surface area analysis of samples

Samples	*S*_BET_ (m^2^ g^−1^)	Pore volume (cm^3^ g^−1^)	Pore size (nm)
Pt_5_Fe_5_/N-CeO_2_	142.827	0.446	9.135
Pt_5_Ni_5_/N-CeO_2_	133.858	0.450	8.079
Pt_5_Zn_5_/N-CeO_2_	129.307	0.408	7.501
Pt_5_Mg_5_/N-CeO_2_	126.315	0.438	6.354

**Fig. 5:**
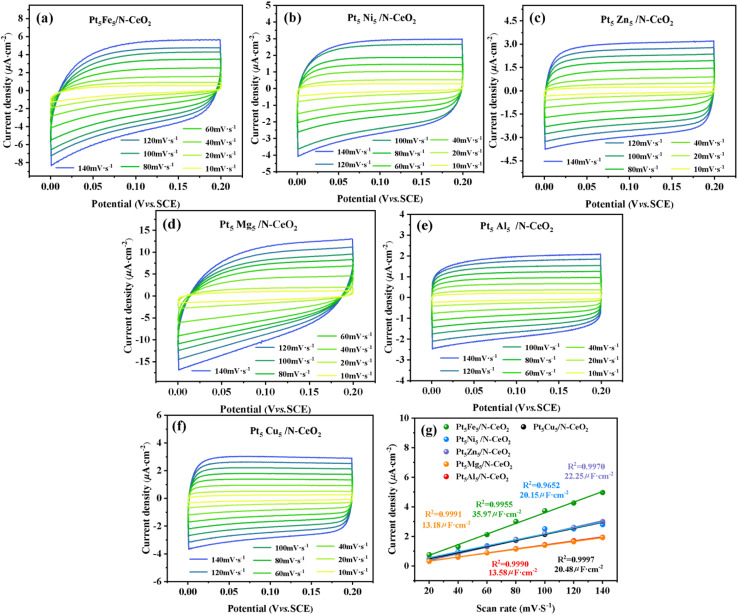
(a–f) CV curves of Pt_5_M_5_/N-CeO_2_ at different scan rates and (g) linear relation between the current density and scan rate of various metal-loaded catalysts.

Fig. 5a–f show the CV curves of Pt_5_Fe_5_/N-CeO_2_, Pt_5_Ni_5_/N-CeO_2_, Pt_5_Zn_5_/N-CeO_2_, Pt_5_Mg_5_/N-CeO_2_, Pt_5_Al_5_/N-CeO_2_ and Pt_5_Cu_5_/N-CeO_2_ at different scanning rates. Fig. 5g presents the relationship between the maximum current density of the sample and the scanning rate. It can be seen that the *C*_dl_ values of the sample are different under different bimetal loads. The *C*_dl_ value of Pt_5_Fe_5_/N-CeO_2_ is 35.97 µF cm^−2^. It is significantly higher than those for Pt_5_Ni_5_/N-CeO_2_ (20.15 µF cm^−2^), Pt_5_Zn_5_/N-CeO_2_ (22.25 µF cm^−2^), Pt_5_Mg_5_/N-CeO_2_ (13.18 µF cm^−2^), Pt_5_Al_5_/N-CeO_2_ (13.58 µF cm^−2^) and Pt_5_Cu_5_/N-CeO_2_ (20.48 µF cm^−2^), indicating that Pt_5_Fe_5_/N-CeO_2_ exposes more active sites than the other samples. The introduction of Fe into the Pt-based catalyst significantly enhances the charge storage capacity compared with other non-precious metal promoters, thereby providing more catalytic sites and facilitating redox kinetics during the catalytic reaction, suggesting that Pt_5_Fe_5_/N-CeO_2_ is more effective in promoting the overall catalytic performance.

**Fig. 6:**
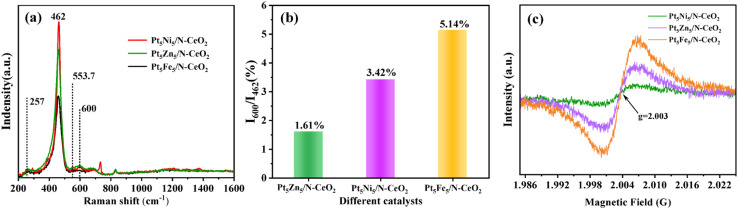
(a) Raman spectra, (b) defect degree characterization, and (c) EPR spectra of Pt_5_Ni_5_/N-CeO_2_, Pt_5_Zn_5_/N-CeO_2_, and Pt_5_Fe_5_/N-CeO_2_.

Defects and oxygen vacancies on the catalyst surface play a key role in the thermal catalytic water reforming of methanol. Raman spectroscopy can be used to characterize the structure of defects and oxygen vacancy concentration in catalysts. As shown in Fig. 6a, the Ce–O–Ce symmetric stretching vibration peak of the F_2g_ mode in the fluorite cubic cerium oxide structure is located at 462 cm^−1^, and its position and shape can be adjusted by changing the microcrystal size. In contrast, the Raman peaks of the F_2g_ mode in the Pt_5_Fe_5_/N-CeO_2_ sample are weaker than those of the other samples, which can be attributed to the sample having the smallest grain size. The weak peak observed at 257 cm^−1^ corresponds to the second-order transverse acoustic mode (2 TA), while another weak peak around 600 cm^−1^ is assigned to the defect induction band of the sample (the “D” band). The intensity can be used to measure the deformation of the anionic lattice, which is responsible for surface defects and oxygen vacancies in the sample.^24,25^ The relatively weak peak at 553.7 cm^−1^ was attributed to oxygen vacancies, and the relative intensity ratio of the D band to the F_2_g mode (*I*_600_/*I*_462_) provides a quantitative measure of defect density and oxygen vacancy concentration. As shown in Fig. 6b, *I*_600_/*I*_462_ of Pt_5_Fe_5_/N-CeO_2_ is higher than those of Pt_5_Ni_5_/N-CeO_2_ and Pt_5_Zn_5_/N-CeO_2_. The results show that Pt_5_Fe_5_/N-CeO_2_ has more structural defects and oxygen vacancy concentration than Pt_5_Ni_5_/N-CeO_2_ and Pt_5_Zn_5_/N-CeO_2_. The Fe-Pt-Ce interactions between the loaded metal and Ce promote the formation of defects and interfacial oxygen vacancies, which is consistent with XRD patterns and TEM results.

Electron paramagnetic resonance (EPR) is an important technique for directly detecting and studying crystal defects, and it provides further insights into the formation of oxygen vacancies in catalysts. As shown in Fig. 6c, Pt_5_Ni_5_/N-CeO_2_, Pt_5_Zn_5_/N-CeO_2_, and Pt_5_Fe_5_/N-CeO_2_ exhibit defect signals at *g* = 2.003, indicating the presence of unpaired electrons associated with oxygen atoms. The intensity of these signals correlates with the concentration of oxygen vacancies.^26,27^ Among the samples, Pt_5_Fe_5_/N-CeO_2_ shows the highest peak intensity, suggesting a higher concentration of oxygen vacancies, which is consistent with the Raman spectroscopy results. The increase in oxygen vacancies leads to a greater number of active surface sites and facilitates their dynamic participation in the reaction cycle. These vacancies interact with adjacent metal species, synergistically enhancing the dehydrogenation reaction.

**Fig. 7:**
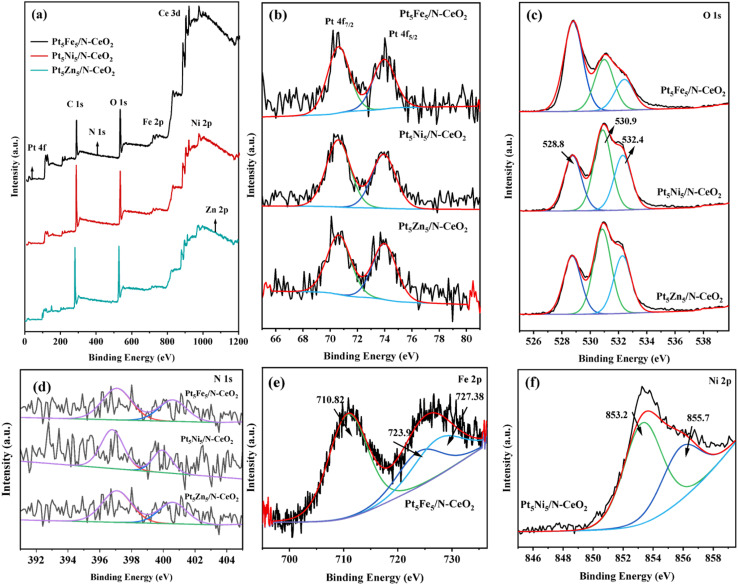
XPS spectra of Pt_5_Fe_5_/N-CeO_2_, Pt_5_Ni_5_/N-CeO_2_ and Pt_5_Zn_5_/N-CeO_2_: (a) survey spectrum and (b) Pt 4f, (c) O 1s, (d) N 1s, (e) Fe 2p, and (f) Ni 2p spectra.

Fig. 7 shows the XPS survey spectra of Pt_5_Fe_5_/N-CeO_2_, Pt_5_Ni_5_/N-CeO_2_ and Pt_5_Zn_5_/N-CeO_2_. It can be observed that the peaks corresponding to N, Zn, and Pt are not clearly visible, which may be attributed to their low content and consequently weak signal intensity. Fig. 7b shows the Pt 4f spectra of the sample. The binding energies at 70.6 eV and 73.8 eV correspond to Pt^2+^ 4f_7/2_ and Pt^0^ 4f_5/2_, respectively. The binding energy of the Pt^0^ 4f_5/2_ orbitals in Pt_5_Fe_5_/N-CeO_2_ is higher than that of Pt_5_Ni_5_/N-CeO_2_ and Pt_5_Zn_5_/N-CeO_2_, indicating a stronger metal–support interaction and a higher proportion of metallic Pt species on the surface of the Pt_5_Fe_5_/N-CeO_2_ catalyst. which is consistent with the results of Raman analysis. Fig. 7c shows the O 1s XPS spectra of the sample. The binding energy peaks at 528.8 eV, 530.9 eV and 532.4 eV are attributed to lattice oxygen, surface adsorbed oxygen and hydroxyl groups or adsorbed water, respectively. It is observed that the lattice oxygen peak of Pt_5_Fe_5_/N-CeO_2_ shifts from 532.4 eV to 532.6 eV, suggesting that the introduction of Fe promotes the activation and migration of lattice oxygen compared with other samples. Fig. 7e shows the Fe 2p spectrum of Pt_5_Fe_5_/N-CeO_2_. Three main peaks at 710.8 eV, 723.9 eV and 727.38 eV are observed, which are attributed to Fe^3+^ and Fe^2+^, corresponding to Fe 2p_3/2_ and Fe 2p_1/2_ orbitals, respectively. This indicates the presence of Fe-Pt-Ce interfacial structures. In the Ni 2p spectrum (Fig. 7f), the peaks at 853.24 eV and 855.7 eV correspond to Ni^0^ and Ni^2+^, respectively.^17^ The N 1s XPS spectra (Fig. 7d) of Pt_5_Fe_5_/N-CeO_2_, Pt_5_Ni_5_/N-CeO_2_ and Pt_5_Zn_5_/N-CeO_2_ show two peaks, corresponding to O–N and pyrrolic N, respectively.

### 3.2 Analysis of the methanol steam reforming reaction on the Pt_*x*_M_10−*x*_/N-CeO_2_ catalysts

**Fig. 8:**
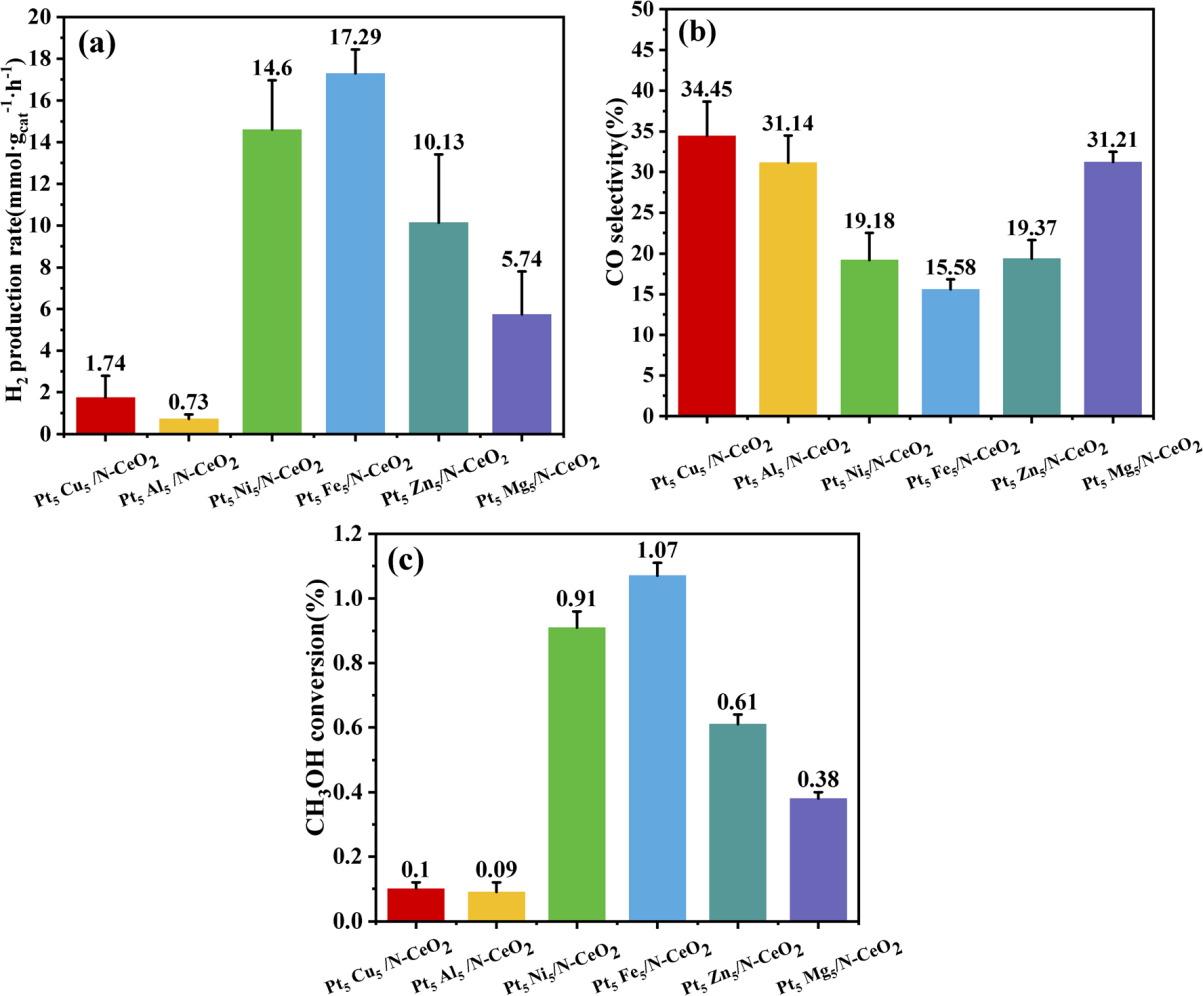
(a) H_2_ gas production rate, (b) CO selectivity, and (c) methanol conversion of various catalysts.

The methanol steam reforming performance of Pt_5_M_5_/N-CeO_2_ was evaluated by measuring the H_2_ production rate, CO selectivity, and methanol conversion. The reaction was carried out in a fixed-bed reactor using 0.2 g of 180–200 mesh catalyst particles mixed with 1 g of 120–180 mesh quartz sand. The temperature was increased to 180 °C at a heating rate of 5 °C min^−1^ under an Ar flow of 35 mL min^−1^. The molar ratio of methanol to water was 1 : 1.2, the space velocity was 30 mL h^−1^ g^−1^, and the feed rate of the methanol–water mixture was maintained at 0.1 mL min^−1^. As shown in Fig. 8, the catalytic performances of all samples are summarized in terms of H_2_ production rate, CO selectivity, and methanol conversion. Fig. 8a presents the H_2_ production rate of the MSR reaction. Among all the bimetallic catalysts, Pt_5_Fe5/N-CeO_2_ exhibits the highest H_2_ production rate (17.29 mmol *g*_cat_^−1^·h^−1^), which is 1.18, 1.71, 3.01, 9.94, and 23.68 times higher than those of Pt_5_Ni_5_/N-CeO_2_ (14.6 mmol *g*_cat_^−1^·h^−1^), Pt_5_Zn_5_/N-CeO_2_ (10.13 mmol *g*_cat_^−1^·h^−1^), Pt_5_Mg_5_/N-CeO_2_ (5.74 mmol *g*_cat_^−1^·h^−1^), Pt_5_Al_5_/N-CeO_2_ (1.74 mmol *g*_cat_^−1^·h^−1^), and Pt_5_Cu_5_/N-CeO_2_ (0.73 mmol *g*_cat_^−1^·h^−1^), respectively. The enhanced activity can be attributed to the role of Fe as an additional active site, which synergistically interacts with Pt and strengthens the metal–support interaction.

Fig. 8b shows the CO selectivity of catalysts during the MSR reaction. The CO selectivity values of Pt_5_Fe_5_/N-CeO_2_, Pt_5_Ni_5_/N-CeO_2_, Pt_5_Zn_5_/N-CeO_2_, Pt_5_Mg_5_/N-CeO_2_, Pt_5_Al_5_/N-CeO_2_, and Pt_5_Cu_5_/N-CeO_2_ are 34.45%, 31.14%, 19.18%, 15.58%, 19.37%, and 31.21%, respectively. Among these, the Fe-containing catalyst exhibits a relatively lower CO selectivity, indicating that Fe plays a positive role in suppressing by-product formation and improving the catalytic performance in the bimetallic system. Fig. 8c illustrates the methanol conversion of each catalyst. Pt_5_Fe_5_/N-CeO_2_ exhibits the highest methanol conversion (1.07%), which is consistent with its superior H_2_ production performance. These results demonstrate that the Pt–Fe bimetallic system exhibits the most favorable interaction with the CeO_2_ support. Notably, this synergy enables a reduced usage of the noble metal Pt while maintaining high catalytic activity, thereby lowering the overall catalytic cost.

### 3.3 Morphology and structure analysis of the Pt_*x*_Fe_10−*x*_/N-CeO_2_ catalysts

**Fig. 9:**
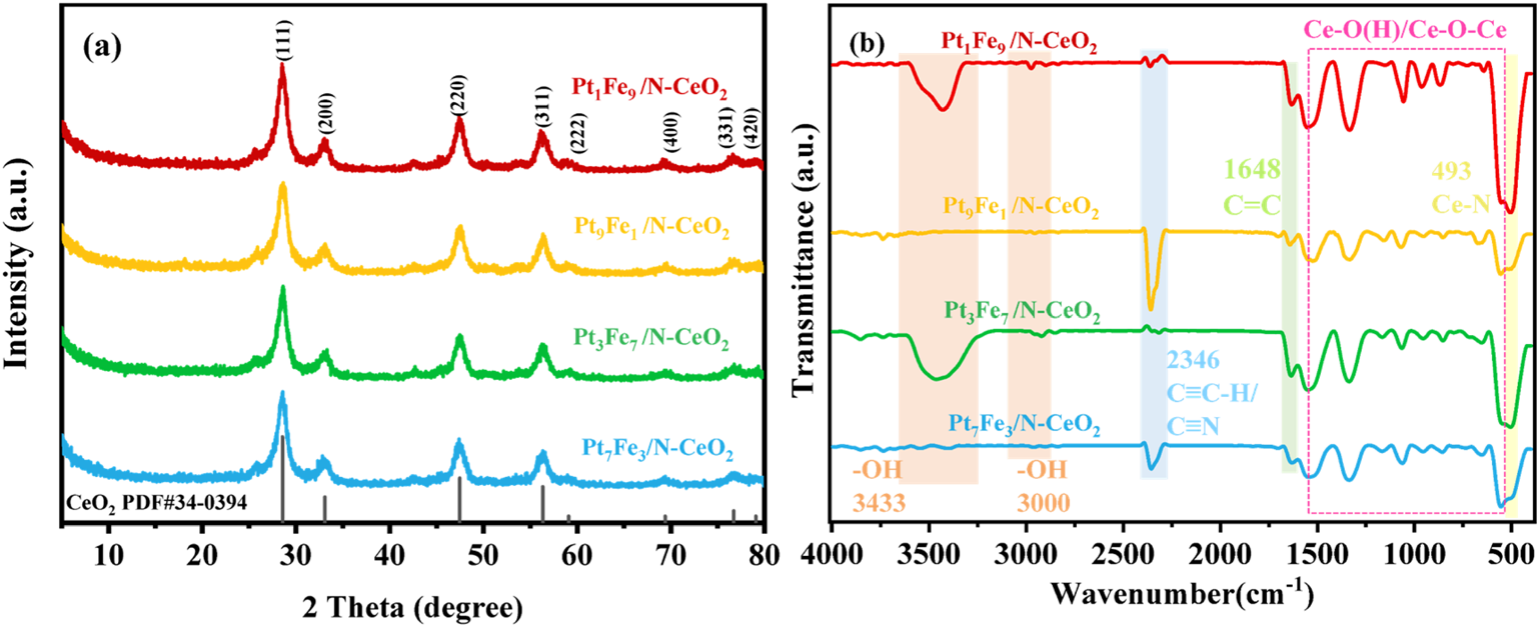
XRD patterns (a) and FT-IR spectra (b) of the Pt_X_Fe_10−X_/N-CeO_2_ samples with bimetallic molar ratios of Pt and Fe at 7 : 3, 3 : 7, 9 : 1 and 1 : 9.

Fig. 9a presents the XRD patterns of Pt_*x*_Fe_10−*x*_/N-CeO_2_ catalysts (where *x* denotes the molar ratio of Pt to Fe). All the samples display characteristic diffraction peaks at 2*θ* = 28.5°, 33.1°, 47.4°, 56.3°, 59.0°, 69.4°, 76.6° and 79.1°, which can be indexed to the (111), (200), (220), (311), (222), (400), (331), and (420) planes of fluorite-structured CeO_2_ (PDF#34-0394). However, no discernible diffraction peaks associated with Pt or Fe species are detected, which can be reasonably ascribed to their ultralow loading, rendering them below the detection limit of XRD. In addition, it can be observed that the diffraction peak intensities of (111), (200), (220), and (311) planes of the Pt_1_Fe_9_/N-CeO_2_ sample are significantly higher than those of the other samples. This may be attributed to the increased Fe content, which could alter the crystallinity of the catalyst or promote the growth of CeO_2_, thereby enhancing the diffraction intensities of (111), (200), (220) and (311) planes.

Fig. 9b shows the FT-IR spectra of the catalysts. It can be observed that the characteristic peak positions of the four samples are similar. The peak near 3433 cm^−1^ corresponds to the O–H stretching vibration of adsorbed water. However, no corresponding peak is observed for Pt_9_Fe_1_/N-CeO_2_ and Pt_7_Fe_3_/N-CeO_2_ in this region, which may be related to the humidity of the ambient air during sample preparation and measurement. The peak at 2346 cm^−1^ is associated with the stretching vibration of triple bonds (C≡C–H) or nitrile groups (C≡N), indicating that the N species are incorporated into the samples and form chemical bonds. The band at 493 cm^−1^ can be attributed to Ce–N vibrations. Meanwhile, the peaks below 1660 cm^−1^ (in the range of 493–1552 cm^−1^) are assigned to Ce–O(H) stretching modes, confirming the presence of the CeO_2_ phase in all the samples. It is notable that the intensity of the peak at 493 cm^−1^ gradually increases with increasing Fe loading, suggesting that the Pt-to-Fe atomic ratio influences the crystal structure and growth behavior of the catalyst.

**Fig. 10:**
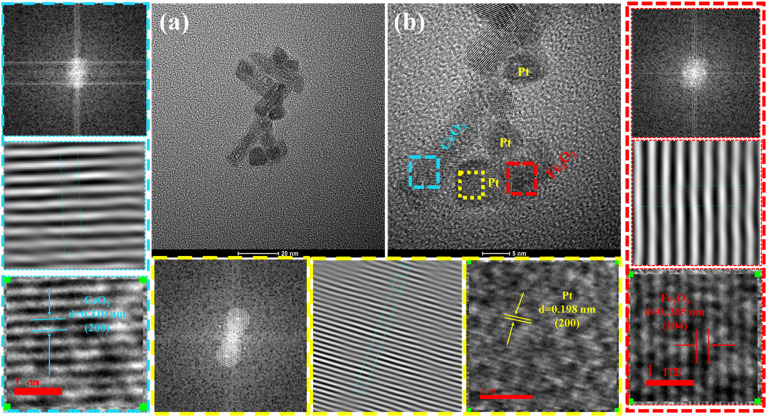
(a and b) TEM images of Pt_5_Fe_5_/N-CeO_2_.

The morphology and microstructure of the Pt_5_Fe_5_/N-CeO_2_ sample were examined by transmission electron microscopy (TEM). As shown in Fig. 10a, the sample exhibits a nanorod structure with a length of approximately 20 nm and an average diameter of about 6 nm. As shown in Fig. 10b, clear lattice fringes can be observed on the CeO_2_ nanorods, with interplanar spacings of 0.310 nm and 0.280 nm, corresponding to the (111) and (200) planes of CeO_2_, respectively. In addition, Pt nanoparticles in close contact with CeO_2_ were observed, with a particle size of about 5 nm. These nanoparticles display distinct lattice fringes with a spacing of 0.198 nm, which can be assigned to the (200) plane of Pt. Furthermore, lattice fringes attributable to Fe_2_O_3_ were also observed, with an interplanar spacing of 0.264 nm, corresponding to the (104) crystal plane.

**Fig. 11:**
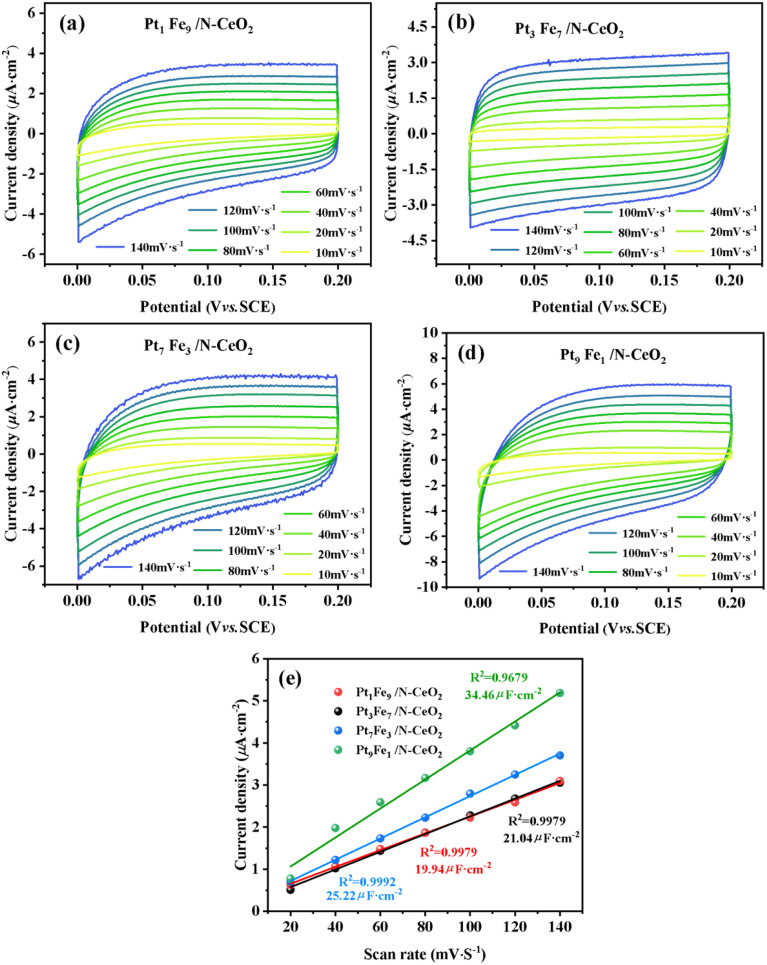
(a–d) CV curves at different scan rates of Pt_X_Fe_10−X_/N-CeO_2_ and (e) linear relation between their current density and scan rate.

Fig. 11a–d present the CV curves of Pt_9_Fe_1_/N-CeO_2_, Pt_3_Fe_7_/N-CeO_2_, Pt_7_Fe_3_/N-CeO_2_, and Pt_1_Fe_9_/N-CeO_2_ at different scan rates. The bimetallic catalysts exhibit distinct current responses, indicating differences in their electrochemical properties. As shown in Fig. 11e, the Pt/Fe molar ratio significantly influences the *C*_dl_ of the samples. The variation can be attributed to changes in particle size and oxygen vacancy concentration, which affect the number of accessible active sites. In addition, the variation in Pt and Fe contents may alter the lattice structure, including porosity and thickness. Among all samples, Pt_9_Fe_1_/N-CeO_2_ exhibits the highest *C*_dl_ value (34.46 µF cm^−2^), which is approximately 1.7 times higher than that of Pt_1_Fe_9_/N-CeO_2_ (19.94 µF cm^−2^), indicating a larger electrochemically active surface area and more exposed active sites. The *C*_dl_ values of Pt_7_Fe_3_/N-CeO_2_ and Pt_3_Fe_7_/N-CeO_2_ are 25.22 and 21.04 µF cm^−2^, respectively. As Pt loading decreases, the *C*_dl_ value gradually declines, which can be attributed to the reduced number of highly active Pt sites.

**Fig. 12:**
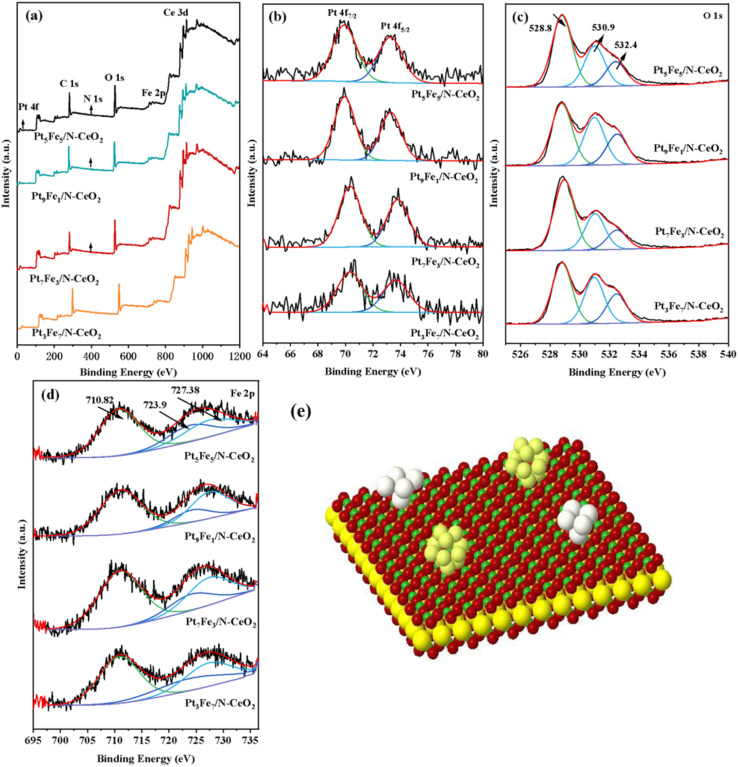
XPS spectra of Pt_X_Fe_10−X_/N-CeO_2_: (a) survey spectrum and (b) Pt 4f, (c) O 1s, and d) Fe 2p spectra. (e) Metal atom-loaded doping structure.

To characterize the chemical states of the elements in the samples, X-ray photoelectron spectroscopy (XPS) was performed on Pt_5_Fe_5_/N-CeO_2_, Pt_9_Fe_1_/N-CeO_2_, Pt_7_Fe_3_/N-CeO_2_, and Pt_3_Fe_7_/N-CeO_2_. Fig. 12a presents the full survey spectra of four samples, showing the characteristic peaks for Ce 3d, Fe 2p, O 1s, N 1s, and C 1s at their corresponding binding energies. Peaks corresponding to Pt and Fe were not clearly observed in the survey spectra, which can be attributed to the low loading levels of these metals. Fig. 12b presents the Pt 4f spectra of Pt_5_Fe_5_/N-CeO_2_, Pt_9_Fe_1_/N-CeO_2_, Pt_7_Fe_3_/N-CeO_2_, and Pt_3_Fe_7_/N-CeO_2_. Metallic Pt serves as the active site for methanol steam reforming, with characteristic binding energies located at approximately 70.6 eV (Pt 4f_7/2_) and 73.8 eV (Pt 4f_5/2_). As Pt is progressively substituted by Fe, the binding energy of Pt shifts to lower values, indicating a modification of its electronic environment due to metal–metal interactions. Fig. 12c shows the O 1s spectra of the samples. The peaks located at 528.8 eV, 530.9 eV, and 532.4 eV are assigned to lattice oxygen, surface-adsorbed oxygen, and hydroxyl groups or adsorbed water species, respectively.^28^ With increasing Fe loading, the O 1s peaks shift toward higher binding energies, with the most pronounced shift observed for Pt_5_Fe_5_/N-CeO_2_. The shift plays a crucial role in facilitating the migration and activation of lattice oxygen species.^29^ Meanwhile, the activation and mobility of lattice oxygen are closely associated with oxygen vacancies on the catalyst surface.^30,31^ The enhanced intensity of the lattice oxygen peak suggests a decrease in electron cloud density, indicating stronger metal–support interactions. Fig. 12d displays the Fe 2p spectra of the samples. Three main peaks at 710.8 eV, 723.9 eV, and 727.38 eV are observed in the Fe 2p spectra, corresponding to Fe^3+^ and Fe^2+^ species in the Fe 2p_3/2_ and Fe 2p_1/2_ orbitals.^32^ Slight shifts in the peaks at 710.8 eV and 723.9 eV for Pt_5_Fe_5_/N-CeO_2_ indicate that the Pt/Fe molar ratio influences the strength of the chemical bonding and electronic interaction between the metal species. Fig. 12e illustrates the three-dimensional crystal structure of Pt_*x*_Fe_10−*x*_/N-CeO_2_. In the model, green represents N dopants on the CeO_2_ support, dark yellow corresponds to Ce atoms, red denotes O atoms, while the metallic sites are shown in silver-white for Fe and pale yellow for Pt.

### 3.4 Analysis of the methanol steam reforming reaction on Pt_*x*_Fe_10−*x*_/N-CeO_2_

**Fig. 13:**
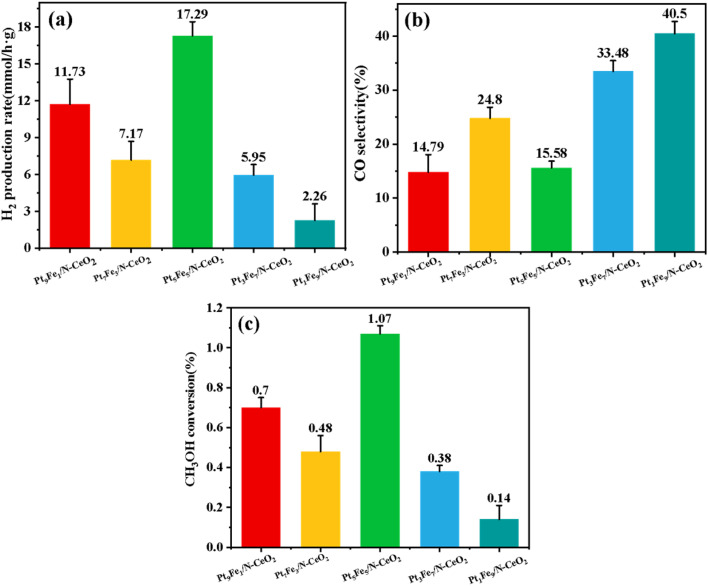
(a) H_2_ production rate, (b) CO selectivity, and (c) methanol conversion rate of various catalysts.

The catalytic performance of Pt_*x*_Fe_10−*x*_/N-CeO_2_ samples with varying Pt-to-Fe molar ratios (Pt_1_Fe_9_/N-CeO_2_, Pt_9_Fe_1_/N-CeO_2_, Pt_5_Fe_5_/N-CeO_2_, Pt_7_Fe_3_/N-CeO_2_, and Pt_3_Fe_7_/N-CeO_2_) in the MSR reaction was systematically investigated. As shown in Fig. 13a–c, the H_2_ production rate, CO selectivity, and methanol conversion were evaluated. Fig. 13a indicates that the Pt_5_Fe_5_/N-CeO_2_ sample exhibited the highest H_2_ production rate among the catalysts. The optimal performance is attributed to the balanced ratio of Fe-Pt-Ce structures, which synergistically enhanced catalytic activity. The H_2_ production rate of Pt_5_Fe_5_/N-CeO_2_ is 1.47, 2.41, 3.01 and 7.65 times higher than that of Pt_9_Fe_1_/N-CeO_2_(11.73 mmol *g*_cat_^−1^·h^−1^), Pt_7_Fe_3_/N-CeO_2_ (7.17 mmol *g*_cat_^−1^·h^−1^), Pt_3_Fe_7_/N-CeO_2_ (5.95 mmol *g*_cat_^−1^·h^−1^), and Pt_1_Fe_9_/N-CeO_2_ (2.26 mmol *g*_cat_^−1^·h^−1^), respectively. These results highlight the critical role of tuning the Pt–Fe molar ratio in improving MSR performance, aligning with recent advancements in the design of bifunctional catalysts for selective hydrogen production. Fig. 13b presents the CO selectivity of catalysts in methanol steam reforming reactions. The CO selectivity of Pt_9_Fe_1_/N-CeO_2_, Pt_7_Fe_3_/N-CeO_2_, Pt_3_Fe_7_/N-CeO_2_ and Pt_1_Fe_9_/N-CeO_2_ is 14.79%, 24.8%, 33.48%, and 40.5%, respectively. Notably, the Pt : Fe molar ratio of 5 : 5 demonstrated the lowest CO selectivity in MSR, indicating that Fe optimally suppresses byproduct formation through bimetallic synergistic regulation. Fig. 13c displays methanol conversion of the samples, where a molar ratio of 5 : 5 exhibited the highest conversion efficiency, consistent with the H_2_ production rate trends. Experimental results suggest that the Pt–Fe (5 : 5) bimetallic system achieves optimal metal–support interactions, facilitating accelerated C–H bond cleavage in methanol and water molecules with the lowest energy barrier (minimum energy level required for activation). The configuration exhibits the highest methanol reforming performance due to favorable interfacial electronic effects and reduced energy barriers. Furthermore, a comparison of catalysts reported for hydrogen production *via* methanol steam reforming is presented in Table 2.

**Table 2:** Comparison of the reaction conditions and catalytic performance of different catalysts for methanol steam reforming

Catalyst	Temperature (°C)	WHSV (h^−1^)	*S*/*C*	X (CH_3_OH) (%)	Y(H_2_) mmol·h^−1^·*g*_cat_^−1^	Ref.
Cu/CeO_2_	250	—	1	66.3	490	33
PdCu/CeO_2_-Al_2_O_3_	250	4	1.5	50	—	34
Cu/ZnO/Al_2_O_3_	200	6	1.3	—	95.5	35
CuO/ZnO	300	30	1.3	98.3	—	36
CuCo_2_O_4_	320	4.32	1.2	100	0.24	37
CuO-ZnO-Al_2_O_3_/CeO_2_	280	6.05	1.2	100	0.672	38
Pt_5_Fe_5_/N-CeO_2_	180	24	1.2	1.08	18.8	This work

**Fig. 14:**
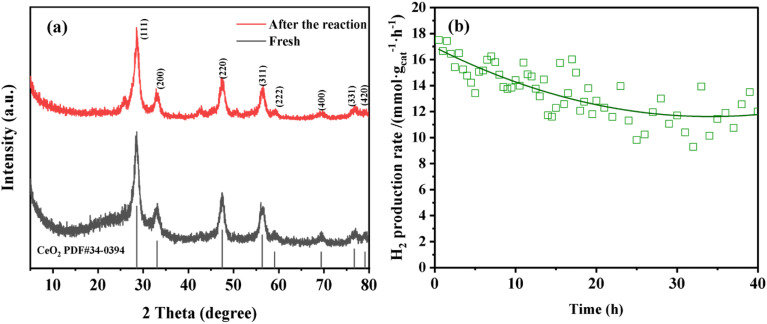
(a) XRD patterns of the Pt_5_Fe_5_/N-CeO_2_ sample after 40 h of methanol steam reforming reaction. (b) Stability evaluation of the Pt_5_Fe_5_/N-CeO_2_ sample in the MSR reaction.

Fig. 14a and b present the stability evaluation results of the Pt_5_Fe_5_/N-CeO_2_ catalyst after 40 h of operation in the methanol steam reforming reaction. The results indicate that the Pt_5_Fe_5_/N-CeO_2_ catalyst maintains good stability after 40 h of continuous reaction. The H_2_ production rate decreases from an initial value of approximately 17 mmol *g*_cat_^−1^·h^−1^ to about 12 mmol *g*_cat_^−1^·h^−1^, suggesting that the catalyst exhibits favorable structural integrity and catalytic stability during the long-term operation.

### 3.5 Analysis of the methanol steam reforming (MSR) reaction kinetics over Pt_*x*_M_10−*x*_/N-CeO_2_-based catalysts

**Fig. 15:**
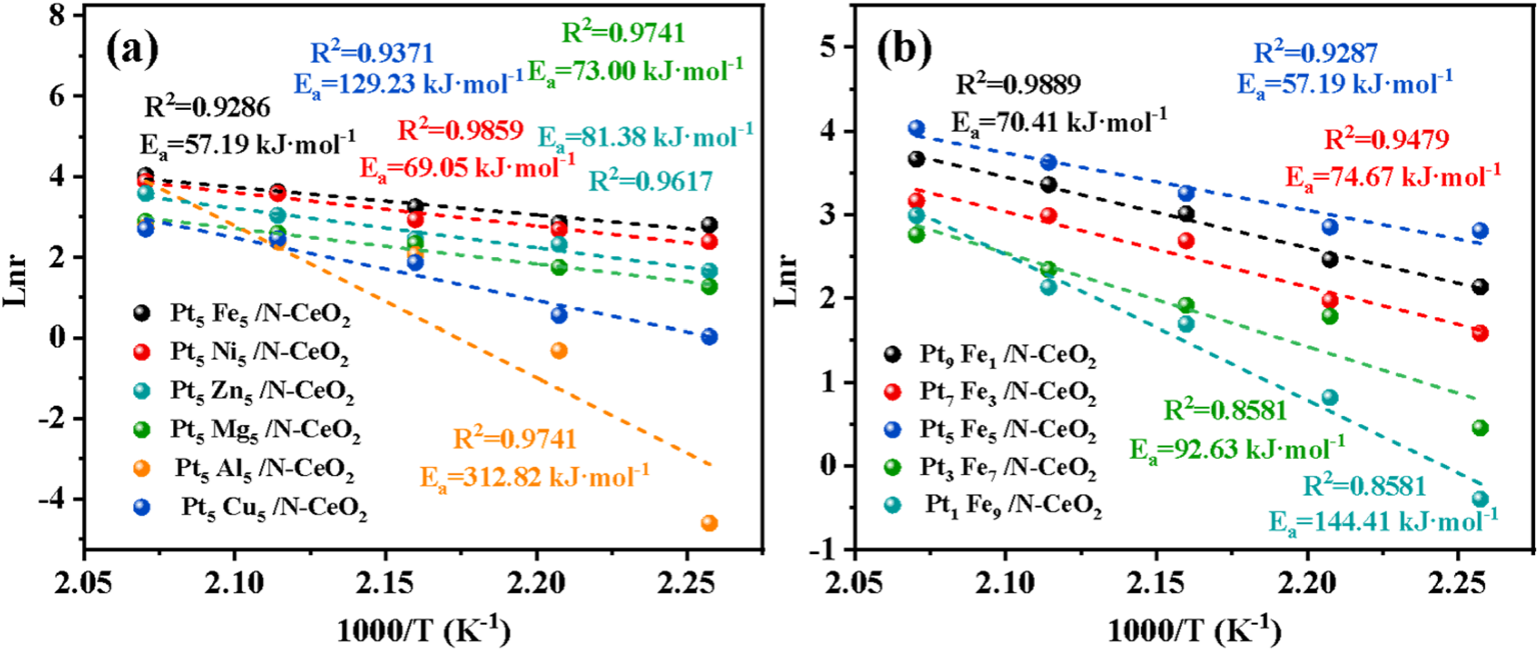
(a and b) Reaction rate and temperature curves of Pt_X_M_10−X_/N-CeO_2_.

Based on the hydrogen production rates of Pt_*x*_M_10−*x*_/N-CeO_2_ at different temperatures Tables 3 and 4, the reaction energy barriers of bimetallic Pt–M (M = Fe, Ni, Zn, Mg, Al, Cu) sites in methanol steam reforming (MSR) were systematically investigated. Fig. 15a displays the Arrhenius plots for Pt_5_Fe_5_/N-CeO_2_, Pt_5_Ni_5_/N-CeO_2_, Pt_5_Zn_5_/N-CeO_2_, Pt_5_Mg_5_/N-CeO_2_, Pt_5_Al_5_/N-CeO_2_, and Pt_5_Cu_5_/N-CeO_2_. The calculated activation energies reveal that Pt_5_Al_5_/N-CeO_2_ exhibits the highest activation energy (312.82 kJ mol^−1^), which is 5.5 times higher than that of Pt_5_Fe_5_/N-CeO_2_ (57.19 kJ mol^−1^) and significantly exceeds those of Pt_5_Ni_5_/N-CeO_2_ (69.05 kJ mol^−1^), Pt_5_Zn_5_/N-CeO_2_ (129.23 kJ mol^−1^), Pt_5_Mg_5_/N-CeO_2_ (73.00 kJ mol^−1^), and Pt_5_Cu_5_/N-CeO_2_ (129.23 kJ mol^−1^), indicating that Fe substitution for Pt provides the lowest energy barrier for MSR and minimizes energy consumption.

**Table 3:** Hydrogen production rates of Pt_*x*_M_10−*x*_/N-CeO_2_ (M = Fe, Ni, Zn, Mg, Al, and Cu) at different temperatures

*T* (°C)	Pt_5_Fe_5_/N-CeO_2_	Pt_5_Ni_5_/N-CeO_2_	Pt_5_Zn_5_/N-CeO_2_	Pt_5_Mg_5_/N-CeO_2_	Pt_5_Al_5_/N-CeO_2_	Pt_5_Cu_5_/N-CeO_2_
170	16.5	10.87	5.22	3.56	0.01	1.03
180	17.29	14.60	10.13	5.74	0.73	1.74
190	25.88	18.74	11.64	10.34	7.88	6.44
200	37.58	35.86	20.89	13.45	10.76	11.67
210	56.42	48.59	35.92	17.94	15.45	14.84

**Table 4:** Hydrogen production rates of Pt_*x*_Fe_10−*x*_/N-CeO_2_ at different temperatures

*T* (°C)	Pt_9_Fe_1_/N-CeO_2_	Pt_7_Fe_3_/N-CeO_2_	Pt_5_Fe_5_/N-CeO_2_	Pt_3_Fe_7_/N-CeO_2_	Pt_1_Fe_9_/N-CeO_2_
170	8.45	4.87	16.5	1.57	0.67
180	11.73	7.17	17.29	5.95	2.26
190	20.32	14.65	25.88	6.79	5.43
200	28.76	19.87	37.58	10.42	8.42
210	38.99	23.67	56.42	15.78	15.45

As shown in Table 5, Pt_5_Fe_5_/N-CeO_2_ also exhibits the lowest pre-exponential factor, suggesting that under these conditions, the sample achieves the optimal collision frequency during the reaction. Consistent with Raman and BET analyses, the observed differences in the apparent activation energy are related to the concentration of surface oxygen species. The presence of oxygen vacancies creates unsaturated active sites, which serve as catalytic centers controlling the reaction rate and reducing the energy barrier. Moreover, C–H bond cleavage is a key rate-determining step in MSR. Experimental results show that, compared with other non-precious metals, Fe loading promotes C–H bond cleavage, which accounts for the superior catalytic activity of the sample.

**Table 5:** Comparison of the kinetic data of samples

Samples	*E*_a_ (kJ mol^−1^)	*A*
Pt_5_Fe_5_/N-CeO_2_	57.19	18.183
Pt_5_Ni_5_/N-CeO_2_	69.05	21.078
Pt_5_Zn_5_/N-CeO_2_	66.26	23.771
Pt_5_Mg_5_/N-CeO_2_	74.74	21.152
Pt_5_Al_5_/N-CeO_2_	312.82	81.795
Pt_5_Cu_5_/N-CeO_2_	129.23	35.123
Pt_9_Fe_1_/N-CeO_2_	70.41	21.236
Pt_7_Fe_3_/N-CeO_2_	74.67	21.895
Pt_3_Fe_7_/N-CeO_2_	92.63	25.939
Pt_1_Fe_9_/N-CeO_2_	144.41	39.000

Based on hydrogen evolution experiments at different temperatures, the reaction energy barriers of Pt–Fe bimetallic catalysts with varying molar ratios in methanol steam reforming (MSR) were systematically investigated. Fig. 15b shows the Arrhenius plots for Pt_1_Fe_9_/N-CeO_2_, Pt_9_Fe_1_/N-CeO_2_, Pt_5_Fe_5_/N-CeO_2_, Pt_7_Fe_3_/N-CeO_2_, and Pt_3_Fe_7_/N-CeO_2_. Calculations revealed that Pt_5_Fe_5_/N-CeO_2_ exhibits the lowest activation energy (57.19 kJ mol^−1^), significantly lower than Pt_9_Fe_1_/N-CeO_2_ (70.41 kJ mol^−1^), Pt_1_Fe_9_/N-CeO_2_ (144.41 kJ mol^−1^), Pt_7_Fe_3_/N-CeO_2_ (74.67 kJ mol^−1^), and Pt_3_Fe_7_/N-CeO_2_ (92.63 kJ mol^−1^). Table 5 demonstrates that increasing the Fe loading enhances the pre-exponential factor, indicating that Fe promotes the reactant-catalyst collision frequency and more Fe sites enhance the collision frequency between the catalyst and reactant molecules. Furthermore, the Pt-to-Fe molar ratio affects the number of active catalytic sites on the surface. When the Pt/Fe molar ratio is 5 : 5, the sample exhibits the lowest energy level for electron transfer and the minimum energy barrier, achieving the most efficient catalytic performance.

### 3.6 Mechanism study of the methanol steam reforming reaction on Pt_*x*_M_10−*x*_/N-CeO_2_-based catalysts

**Fig. 16:**
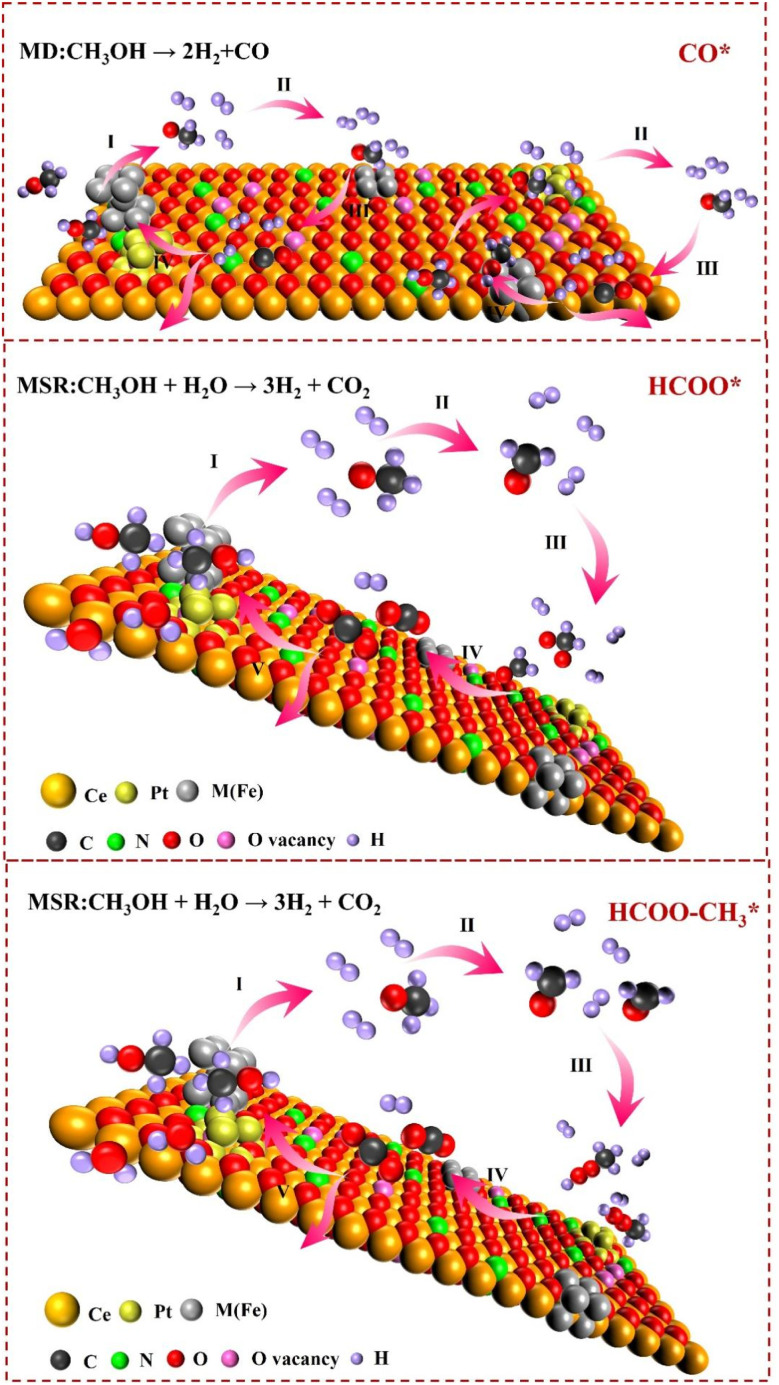
Mechanism of the methanol steam reforming reaction over Pt_*x*_M_10−*x*_/N-CeO_2_.

Based on the above experimental results, the possible reaction pathways for methanol–water reforming on Pt_*x*_M_10−*x*_/N-CeO_2_ are proposed: (i) CH_3_OH spontaneously decomposes into CO and H_2_. Part of the produced CO reacts with water vapor to form CO_2_ and H_2_, while the remaining CO remains in the reaction vessel and waits to react with the remaining water vapor or is discharged from the vessel (denoted as the CO* pathway); (ii) CH_3_OH is further oxidized by the active oxygen generated by hydroxyl groups or H_2_O to form HCOO*, and the intermediate product HCOO* decomposes to produce CO_2_ and H_2_ (denoted as the HCOO* pathway);^39^ and (iii) CH_3_OH undergoes dehydrogenation to form HCOO–CH_3_, which then hydrolyzes to form HCOO*, and subsequently decomposes to produce CO_2_ and H_2_ (denoted as the 
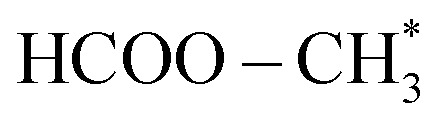
 pathway).^40,41^ Due to the low surface density of Pt and M atoms, gaseous methanol molecules first react with lattice oxygen or surface hydroxyl groups of the N-CeO_2_ support to form top and bridging methoxy species. The N-CeO_2_ support itself exhibits negligible methanol decomposition activity, and the methoxy species must diffuse to the Pt and M sites for further reaction. Among these, the top methoxy species is more reactive than the bridging methoxy species. Considering that these pathways occur almost simultaneously, a sequential MSR reaction mechanism over Pt_*x*_M_10−*x*_/N-CeO_2_ is proposed (Fig. 16). Methanol dehydrogenation occurs at the Pt metal sites, releasing hydrogen and generating CO intermediates. The adjacent oxygen vacancies facilitate the dissociation and adsorption of gaseous methanol. The introduction of M atoms provides additional metal active sites and promotes the formation of oxygen vacancies near the cations, enhancing the reaction of hydroxyl species adsorbed on these vacancies with nearby methanol molecules, thereby minimizing the diffusion limitation of intermediates. Among the possible methanol–water reforming pathways, the CO* pathway is a key factor contributing to catalyst deactivation. Based on the evaluation of catalytic activity with varying bimetallic loadings and atomic ratios, partially substituting Pt sites with Fe appears beneficial for suppressing CO formation. This strategy increases the number of exposed active sites and enhances the overall catalytic activity in methanol steam reforming.

## 4 Conclusion

In this study, a bimetallic cerium-based catalyst (Pt_*x*_M_10−*x*_/N-CeO_2_) was prepared using the high-temperature calcination method. Based on the performance evaluation of MSR, the optimal bimetallic catalyst was found to be Pt_5_Fe_5_/N-CeO_2_. Subsequently, the influence of the Pt-to-Fe atomic ratio on MSR performance was investigated. The results indicate that loading different bimetals can increase the specific surface area, surface defects, and reactive active sites of CeO_2_. XPS testing revealed that the loading of Pt and Fe on the N-CeO_2_ support promotes the migration of lattice oxygen species. The results of the methanol steam reforming experiments show that Pt_5_Fe_5_/N-CeO_2_ exhibits the highest H_2_ production rate and the lowest CO selectivity, with a H_2_ production rate of 17.29 mmol *g*_cat_^−1^·h^−1^, CO selectivity of 15.58%, and methanol conversion rate of 1.07%, demonstrating the best catalytic performance. Compared with Pt_*x*_Fe_10−*x*_/N-CeO_2_, the H_2_ production rate of Pt_5_Fe_5_/N-CeO_2_ increases, indicating that the difference in activity evaluation is not significant and the cost is reduced while maintaining the catalytic performance. According to the Arrhenius equation, Pt_5_Fe_5_/N-CeO_2_ has the lowest apparent activation energy, suggesting that the sample requires the lowest energy to overcome in the MSR reaction and the reaction occurs to the greatest extent.

## Conflicts of interest

There are no conflicts of interest to declare.

## Data Availability

All data included in this study are available upon request to the corresponding author.
